# Good mid- to long-term results of the cemented oxford phase 3 unicompartmental knee arthroplasty in a non-designer centre

**DOI:** 10.1007/s00167-021-06665-x

**Published:** 2021-07-12

**Authors:** D. M. Moore, G. A. Sheridan, A. Welch-Phillips, J. M. O’Byrne, P. Kenny

**Affiliations:** Department of Orthopaedic Surgery, National Orthopaedic Hospital Cappagh, Cappagh Rd, Cappoge, Dublin, D11 EV29 Ireland

**Keywords:** UKA, Unicompartmental knee arthroplasty, Cement, Survival, Long term, Revision

## Abstract

**Purpose:**

Unicompartmental knee arthroplasty (UKA) provides patients with an alternative treatment to TKA in isolated medial compartment osteoarthritis providing better functional outcomes and faster recovery in the short term. Our aim was to quantify revision rates, predictors of revision, mortality rate and functionality of the Oxford Phase 3 UKA in a non-designer institution.

**Methods:**

This was a retrospective review of prospectively collected regional registry data. All Oxford Phase 3 UKAs performed for medial tibio-femoral osteoarthritis of the knee joint were included from a single academic institution between the period of January 1st 2006 and December 30th 2009. Kaplan-Meier survivorship curves adjusting for loss to follow-up and deceased patients were generated. Primary outcome variables included all-cause and aseptic revision. Secondary outcome variables included functional outcome scores. Patients were reviewed at 6 months, 2 years, 5 years, 10 years and 15 years.

**Results:**

A total of 64 cemented Oxford phase 3 UKAs were performed between January 2006 and November 2009. Fifteen-year follow-up data were available for 51 patients, of these 12 required revision. Survival rates, adjusting for patients that were either lost to follow-up or deceased, were 87.5% at 5 years, 81.4% at 10 years and 76.4% at 15 years. The overall aseptic revision rate at the time of review was 18.75% (*n* = 12). The only significant predictor of postoperative WOMAC score at 15 years was the preoperative WOMAC score (*p* = 0.03).

**Conclusion:**

The Oxford Phase 3 UKA for medial tibio-femoral arthritis has promising outcomes at 15-year follow-up with a survival rate of 76.4% in a non-designer centre.

**Level of Evidence:**

III.

## Introduction

Unicompartmental knee arthroplasty (UKA) provides better functional outcomes and faster recovery in the short term when compared to total knee arthroplasty (TKA) [1–4] thus it is an attractive alternative. It also has the additional benefits of preserving native bone, reduced length of stay, superior cost effectiveness, smaller incision and surgical morbidity in terms of blood loss, hospital stay, satisfaction scores, decreased mortality, better range of motion and proprioception [2, 5–8].

There is a limited amount of literature on the long-term clinical UKA outcomes leading some surgeons to avoid implementing UKA in their practise. Despite promising reports to date regarding quality of life and performance, there remains concern about revision rates and long-term outcomes with UKA as registry data have shown an increased revision rate with UKA when compared with TKA and most studies only reporting 10-year outcomes primarily from designer centres [7, 9–11]. The initial enthusiasm surrounding UKA has been superseded by concern over long-term survival. The conflicting data reported to date on long-term outcomes prompted this analysis of the long-term clinical outcomes of UKA at our centre using the Oxford Phase 3 UKA.

This is a retrospective study of a prospectively recorded regional registry on the mid-long-term survivorship of the the Oxford Phase 3 UKA at a non-designer centre. Our main aim was to quantify revision rates, predictors of revision and functionality using the WOMAC score at a Non-Designer Centre with 15-year follow-up. We hypothesized that revision rates may be higher than those procedures carried out at a non-designer centre.

## Methods

This was a retrospective review of prospectively collected regional registry data. All Oxford Phase 3 UKAs performed for medial tibio-femoral osteoarthritis of the knee were included from a single academic institution between January 1st 2006 and the 30th of December 2009. No power analysis was performed because all eligible patients that were available for inclusion were included in the analysis. Sample size was dependent on the volume of procedures performed within the study time frame. All patients underwent minimally invasive surgery with a medial approach using the cemented Oxford phase 3 meniscal bearing unicondylar prosthesis (*Oxford Partial Knee, Biomet UK Limited, Bridgend, United Kingdom*). Surgical procedures were carried out by consultant surgeons with at least 10-year experience. Institutional Review Board approval was obtained and all patients gave their informed consent to their information being used in the joint register in accordance with Declaration of Helsinki before their inclusion in the study.

Inclusion criteria were the presence of isolated medial knee pain with radiographic evidence of medial compartment degeneration only (Outerbridge Grade IV/bone-on-bone osteoarthritis), a stable joint on examination and a correctable varus deformity (if present). Anyone with bi- or tricompartmental OA was excluded.

Primary outcome measures included all-cause and aseptic revision. Secondary outcomes included functional outcome scores. Pre-operatively, the Western Ontario and McMaster Universities Osteoarthritis Index (WOMAC) scores (which asses five items for pain, two for stiffness, and 17 for functional limitation [12], range of motion and pain scores) were assessed. The WOMAC is a set of standardized questionnaires used to evaluate the severity of osteoarthritis involving the knee and hip, it ranges from 96 to 0 [12]. Following surgery, all patients were followed via the regional joint registry with clinical review assessing WOMAC scores at 6 months, 2 years, 5 years, 10 years and 15 years post-surgery along with any complications or revision underwent.

### Follow-up

Patients were seen in the outpatient clinic and subsequently followed via joint registry review at 6 months, 2, 5, 10 and 15 years, respectively where they underwent repeat radiographs and functional assessment using the WOMAC score. They were also contacted via telephone at these same time intervals by an arthroplasty clinical nurse specialist to update their records noting any complications or revisions. If patients were deceased during the 15-year period, their next of kin or general practitioner was contacted to ascertain the cause of death.

### Statistical analysis

Descriptive statistics were used to describe demographic data. All outcomes were reported with mean values and standard deviations (SD). Categorical predictor and outcome variable relationships were assessed using the fisher’s exact test or the chi-squared (χ^2^) test (if the number of subjects were greater than five per group). Two-sample *t* test with equal variances was used for categorical predictors with interval outcome measures. Simple logistic regression was used to analyse the relationships between two interval variables. Kaplan–Meier curves adjusting for patients that were either deceased or lost to follow-up were generated to illustrate survivorship at 5, 10 and 15 years. A *p* value of less than 0.05 was considered to be statistically significant. Statistical analysis was performed using Stata/IC 13.1 for Mac (64-bit Intel).

## Results

### Demographics

A total of 64 consecutive cemented Oxford phase 3 UKAs were performed between January 1st 2006 and December 1st 2009. There were 38 females (59.4%) in total. The mean age at surgery was 66.4 (SD = 9.31, range 46–85) years. The mean body mass index was 31 (SD = 4.9, range 25–39). All 64 patients had isolated medial compartment osteoarthritis. Table 1 shows the baseline characteristics of the study population.

**Table 1:** Baseline characteristics

Sex [no. (%)]	
F	38 (59.4%)
M	26 (40.6%)
BMI [Mean (S.D)]	31.2 (SD 4.9)
ASA Grade [no. (%)]	
Class 0	40 (78.4%)
Class 1	1 (1.9%)
Class 2	8 (15.7%)
Class 3	2 (3.9%)
Age at surgery [Mean (S.D)]	66.4 (SD 9.3)

### Survivorship

**Fig.1:**
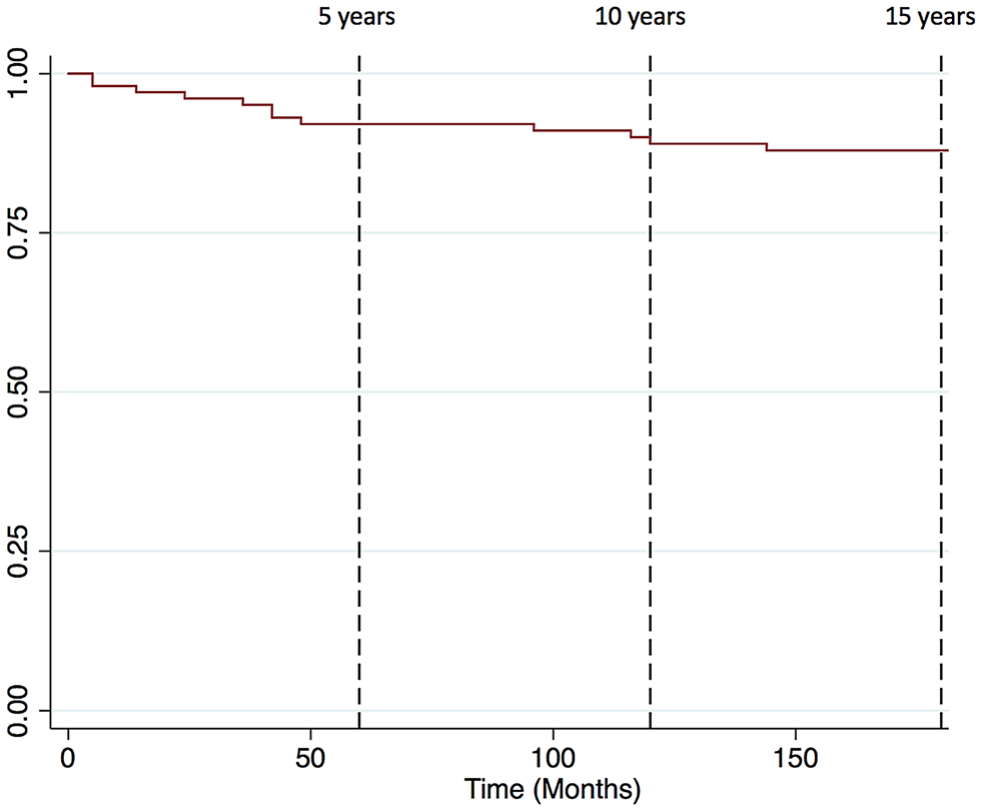
Kaplan-Meier survivorship curve (adjusting for loss to follow-up and death)

Implant survival rates, adjusting for patients that were either lost to follow-up or deceased, were 87.5% at 5 years, 81.4% at 10 years and 76.4% at 15 years (Fig. 1) There were 3 patients deceased at 5 years, 10 deceased at 10 years and 13 deceased at 15 years.

A total of 12 revisions (18.8%) were performed at the time of review. The majority of revisions were for ongoing pain of unknown origin (*n* = 8, 66.7%). Aseptic loosening accounted for 25% (*n* = 3) of revisions. One required revision due to progression of disease. The mean time to revision was 165.2 [± 55.9] weeks. The only significant predictor variable for revision was age with a mean age of 59.2 (SD = 11.46, range 46–78) in the revised group and a mean age of 67.9 (SD = 8.1, range 54–85) in the group that did not require revision (*p* = 0.0072). Other variables such as gender (n.s), BMI (n.s) and ASA grade (n.s) did not have a significant impact revision rates. There was no documented prosthetic joint infection in any case. Of note, two patients required injections for ongoing pain at 5 and 6 years, respectively, both had satisfactory outcomes at final follow-up of 15 years.

### Function

Mean pre-operative and post-operative WOMAC scores are demonstrated in Table 2. The only significant predictor of postoperative WOMAC score at 15 years was preoperative WOMAC score (*p* = 0.03).

**Table 2:** WOMAC score

	Mean	Std. dev	Min	Max
Pre-surgery	48.4	16.9	7	85
6 months post-op	24.5	14.5	1	63
2 years post-op	26.9	18.5	1	73
5 years post-op	23.8	18.7	1	65
15 years post-op	22.1	20.1	0	51

## Discussion

The main finding of this study is that the Oxford Phase 3 medial UKA has an all-cause implant survival rate of 76.4% at 15year follow-up when adjusting for patients either lost to follow-up or deceased. The only significant predictor of revision was young age. Some studies with long-term follow-up showed higher survival rates such as Gupta et al. who reported a 91.86% survival however this included both cemented and uncemented UKAs [13]. They also found that the risk of revision was 1.8-fold higher in the cemented cohort compared to the uncemented cohort [14–16]. This current cohort presented is fully cemented for all cases. The optimal mode of implant fixation in primary UKA is a source of ongoing debate. Whilst reports on short-term implant survival have been positive, long-term registry data have shown a concerning incidence of revision usually as a result of pain or aseptic loosening of the implant [17, 18]. Whilst similar in design, cemented Oxford phase 3 UKA’s have higher mid- to long-term revision rates than uncemented implants [19–21]. Pandit et al. reported a 10 and 15 year implant survival of 96% [16] and 91% [22], respectively, Murray et al. reported a 98% implant survival at 10 year follow-up [23]. Lisowski et al. showed 90.6% 10-year survival in-line with Heaps et al. (91.3%) [24, 25] and British joint registries (90%) [20]. Despite promising reports to date regarding quality of life and performance, there remains concern around high revision rates and long-term implant survival [7].

Mannan et al. reported a 15-year survival rate of 87.8% in populations under 60 [26].

Survival at 15 years for cohorts has been shown to be 87% compared to registries at 69.6% respectively showing a dramatic discrepancy [27]. Australian database of UKAs contains the largest population with the longest follow-up showing a 79% survival rate at 15 years with indication for revision being aseptic loosening (43.5%), osteoarthritis progression (29.4%) and pain (9.5%) [19]. This differs to our finding of pain accounting for the largest reason for revision in 66.7% of patients, however, our rate of revision was shown to be the same at long-term follow-up.

The main reason for revision in our study was pain for 66.7% of revisions, with aseptic loosening (25%) and progression of osteoarthritis (8.3%) being the other indications. Whilst our sample numbers are small in comparison, Gupta et al. reported pain (29.5%) as causing the highest amount of revisions in their analysis of 590 cemented UKA’s [13]. We suspect that the high number of cases revised for ‘pain of unknown origin’ may in fact have been due to progression of the osteoarthritic process elsewhere in the knee joint or else an aseptic loosening that may not have been fully appreciated at the time of revision. Berger et al. found that patella-femoral joint osteoarthritis was the main reason for implant failure at 15-years follow-up [28]. Although Berger et al. analysed fixed bearing implants only, this may be a consideration when interpreting the findings of the current study.

The mean WOMAC functional score at 15 years in the current study was 22. This is a reduction from a mean WOMAC of 48.4. Although not optimal, it still indicates a WOMAC functional score of half that of the pre-op score which we deem to be acceptable at 15 years after surgery. Whilst the WOMAC is not a satisfaction score in itself, this finding correlates with Felts et al. who reported 94% satisfaction for fixed-bearing UKA at 11-year follow-up [29]. When compared with TKA, range of motion and activity scores have been shown to be significantly better in UKA [1]. Previous studies have shown that postoperative patient satisfaction scores depend on preoperative scores along with patient expectation [30, 31]. Our study has shown that good pre-operative WOMAC scores are also the only predictor of good post-operative functional outcomes. BMI and ASA grade did not have a bearing on revision rates or functionality. Interestingly, young age was significantly predictive of revision rates. This may be due to the increased levels of postoperative activity in younger patients which may the in turn accelerate the need for conversion to TKA.

The strength of this study lies in the length of follow-up to 15 years with no surviving patient being lost to follow-up. This information is useful for surgeon and patients alike and may even be used to counsel patients regarding their postoperative functional and implant survival expectations.

### Limitations

This was a single-centre, multi-surgeon study analysing data from one registry. This was also a retrospective study which introduces all of the risks associated with such a study design. We did not report on clinical examination or radiographic findings and so we are limited to conclusions based on the collected data only.

## Conclusion

The Oxford Phase 3 UKA for medial tibio-femoral arthritis has promising outcomes at 15-year follow-up up with a survival rate of 76.4% in a non-designer centre.
